# Large area growth of few-layer In_2_Te_3_ films by chemical vapor deposition and its magnetoresistance properties

**DOI:** 10.1038/s41598-019-47520-x

**Published:** 2019-07-29

**Authors:** Shaohui Zhang, Jingyang Zhang, Baosheng Liu, Xiaobo Jia, Guofu Wang, Haixin Chang

**Affiliations:** 1 Center for Materials Science and Engineering, School of Electrical and Information Engineering, Guangxi University of Science and Technology, Liuzhou, 545006 China; 2 Harbin Institute of Technology, Harbin, 150001 China; 3 State Key Laboratory of Material Processing and Die & Mould Technology, School of Materials Science and Engineering, Huazhong University of Science and Technology, Wuhan, 430074 China

**Keywords:** Two-dimensional materials, Electronic devices

## Abstract

In this work we report a facile route to grow large area, uniform, continuous and few-layer α-In_2_Te_3_ film via chemical vapor deposition (CVD) methods. The characterizations show the large area of CVD-grown few-layer α-In_2_Te_3_. This method guarantees the precise control of thickness down to few layers and large area preparation. The magnetoresistance (MR) properties of few-layer In_2_Te_3_ was investigated from 2 to 300 K and its MR stability under long exposure to ambient air was studied for the first time. Few-layer of α-In_2_Te_3_ shows a positive MR and the largest transverse MR was observed to about 11% at 2 K and a high stability of MR to long time exposure in air up to 21 weeks.

## Introduction

During the past few decades, atomically-thin two-dimensional (2D) materials have attracted tremendous attention because of their dimensionality, like the elemental composition, plays a significant role in their electronic, optical and mechanical properties^1–3^. The discovery of graphene with its astonishing properties and the prediction about Van der Walls heterostructures of atomic layers have led to prosperity of other 2D materials^4,5^, such as transition metal dichalcogenides (TMDs, e.g., MoS_2_, WSe_2_)^6,7^ and compound of group III–V^8^. However, few investigations have been carried out on other 2D layered materials such as III–VI group layered 2D semiconductors that have direct and wide band gaps^9^.

A family of group III–VI semiconductors compound have an enormous potential in fast and sensitive photodetection^10^, optical microcavity^11^ and low-cost semiconductor solar cells^12,13^ applications. The advantages of direct-bandgap 2D III–VI layered semiconductors include high optical-absorption coefficient, light emission and high carrier mobility, and these properties do not require down to monolayer^14^. Thus far, a few investigations have been conducted about indium selenide^15,16^ (InSe and In_2_Se_3_), GaTe^17^, GaSe^18,19^, which are regarded as promising optoelectronic material having an excellent performance in photodetection.

However, indium tellurides, a typical member of group III–VI semiconductors compounds, have not received much intention yet, though it possess the smallest direct band gap (~1.0 eV) value in the known III−VI compounds^20,21^. The various stoichiometric proportions of indium telluride principally including In_2_Te_3_, InTe, In_3_Te_4_, In_4_Te_3_ and In_10_Te_7_ make the facile controlled growth of 2D indium telluride more difficult than other 2D semiconductors^22^. In_2_Te_3_ is more stable and it exhibits two crystalline phases. The disordered β-In_2_Te_3_ exists only in high temperature with a zincblende structure^23^, while α-In_2_Te_3_ has an anti-fluorite structure in low temperature^24^ that possesses a high absorption coefficient exceeding 10^5^ cm^−1 ^^25^. Based on above-mentioned features, this binary semiconductor is an excellent candidate material for using as a photodetector^26^ and also in phase-change random access memory (PRAM)^27^. The traditional techniques for the deposition of In_2_Te_3_ thin films include flash evaporation^28^, thermal evaporation^23,25,29^, vacuum evaporation^22^ and pulsed-laser deposition^26^, and most of the films have thickness of over 200 nm. To prepare large area, atomically-thin few layer α-In_2_Te_3_ films is still a big challenge.

Herein we introduce a facile growth of large area, ultrathin few-layer α-In_2_Te_3_ films via chemical vapour deposition (CVD) methods. This method guarantees the precise control of thickness down to few atomic layers and large area preparation. In addition, we explored the magnetoresistance (MR) properties of few-layer In_2_Te_3_ films which have been little studied before in 2D III–VI layered semiconductors. The magnetoresistance of few-layer In_2_Te_3_ are systematically investigated from 2 to 300 K and its MR stability was studied for the first time under the exposure to air. A positive MR performance was observed that the largest transverse MR value was calculated up to 11% at 2 K and it indicates a high stability of MR under long time exposure in air.

**Figure 1:**
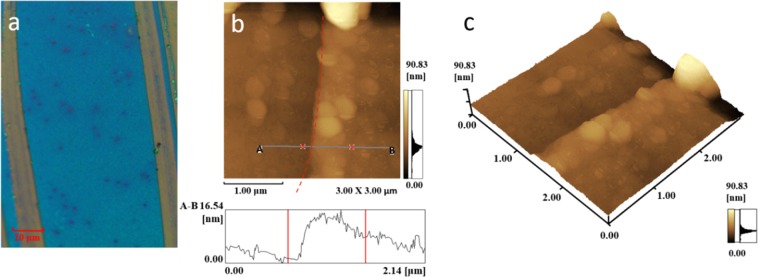
Large area, few-layer In_2_Te_3_ films by CVD. Optical image (**a**) and AFM images (**b**,**c**). The height profile in (**b**) shows a thickness of 6.6 nm.

Using CVD with suitable optimization of temperature and the mass of Te sources, we obtained the large area, few-layer α-In_2_Te_3_ films. Large area and continuous few-layer films were grown only in certain position of the oven and under optimized experimental parameters. The optical microscope images of few-layer In_2_Te_3_ thin films are shown in Fig. 1a. These sample of films have large area with continuity up to centimetre scale. The scratch in Fig. 1a shows the typical contrast discrepancy between few-layer In_2_Te_3_ and SiO_2_/Si substrate which indicates a continuous film, while darker areas in the films displayed a few thicker In_2_Te_3_ crystals which are grown on the surface of the films. AFM was used to determine the thickness of films as shown in Fig. 1b,c. The height profile shows the thickness of few-layer In_2_Te_3_ film is about 6.6 nm (Fig. 1b), indicating a few-layer one.

**Figure 2:**
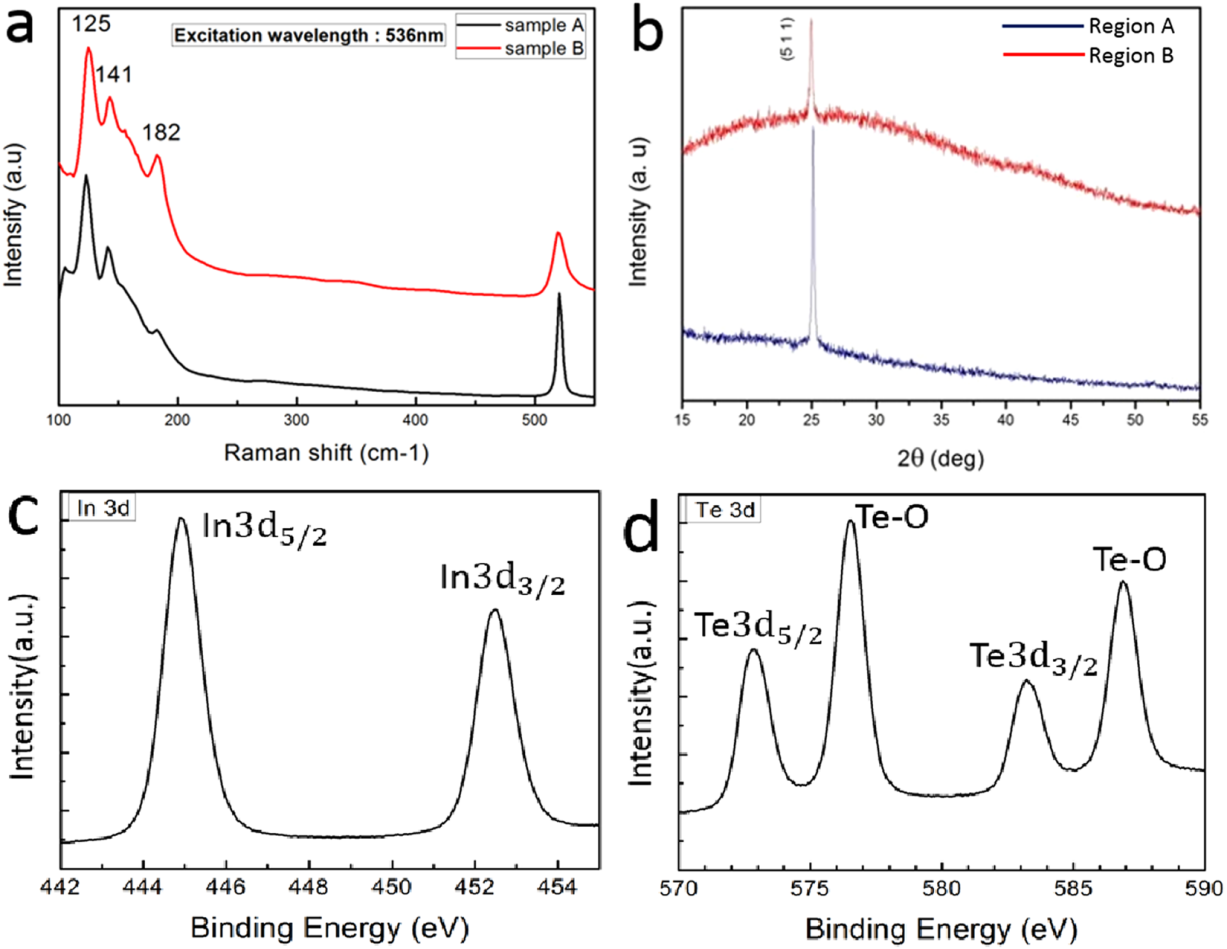
(**a**) Raman spectra from two different few-layer α-In_2_Te_3_ both with typical three Raman peaks at 125, 141 and 182 cm^−1^; (**b**) X-ray diffraction result from a typical α-In_2_Te_3_ film from different spots in the same sample. XPS spectra of (**c**) In 3d and (**d**) Te 3d levels in few-layer α-In_2_Te_3_.

The Raman spectra was obtained from typical few-layer In_2_Te_3_ film under a 532 nm excitation laser and showed peaks at 125, 141 and 182 cm^−1^ (Fig. 2a). Two active modes were shown at 125 and 141 cm^−1^ and that can be assigned to the Te–Te vibration mode in ordered indium telluride^30,31^. Raman shift positions are also highly consistent with the Raman peaks of In_2_Te_3_^26^ that were distinguished from InTe^32,33^. Another weaker peak at 182 cm^−1^ was attributed to the presence of TeO_2_ which was induced by oxidization^34^. XRD was used to study the crystal structures and composition of product. The XRD patterns in Fig. 2b display a slight difference in the intensities of the (511) lattice plane between two regions of the same sample, which implied that the slight difference in crystalline direction and crystallinity in the formed In_2_Te_3_ polycrystalline structures as confirmed by the following TEM imaging^23^. One strong peak can be observed at 25.0° that indicates the diffraction at (511) lattice plane of α-In_2_Te_3_ (JCPDS 33−1488)^35^ and it agreed well with previous reports^25,26,29^. The results of XRD and Raman investigations are highly consistent that indicates the successful preparation of few-layer α-In_2_Te_3_.

XPS spectra are further applied to confirm the chemical composition of the products (Fig. 2c,d**)**. The presence of In, Te and oxidization induced O were observed on XPS survey spectrum of several samples. We measure the evolution of In 3d and Te 3d core levels. Figure 2c shows the binding energy of In 3d_5/2_ located at 445.14 eV^36^ and it’s another peak of In 3d_3/2_ were appeared at 452.48 eV. Figure 2d shows the binding energy of Te 3d_5/2_ and 3d_3/2_ are at 576.95 eV and 586.88 eV respectively, which coincided with previous work^37^. Moreover, a couple of Te-O peaks appeared at 576.79 eV for Te 3d_5/2_ and 586.94 eV for Te 3d_3/2_ respectively that attributed to the oxidation of tellurium including Te^4+^ (TeO_2_) and Te^6+^ (TeO_3_)^25,34^. The presence of O in few-layer α-In_2_Te_3_ suggesting that our α-In_2_Te_3_ surface is highly chemically unstable and easily oxidized to form an ln_2_Te_3_-TeO_x_ surface layer at ambient conditions. Fortunately, the formed ultrathin natural amorphous TeO_x_ surface layer have an approximate self-limiting thickness to limit the further oxidation^38^. Also, the induced surface natural oxide layer function can be used as dielectrics beneficial for potential applications, but impart a possible detrimental effect on transport properties. The XPS results for In 3d and Te 3d core levels are also consistent with reported values for In_2_Te_3_^39^. More importantly, the peak areas in the same orbital peak (both in the 3d orbital) of XPS could semi-quantitative calculate the ratio of corresponding valence states ions^40^. Therefore, it can be calculated that the content ratio of Te: In is 2.85:2 according to the peak areas of Te 3d and In 3d. So the stoichiometric ratio of In and Te elements in the sample approximately consisted with the In_2_Te_3_.

**Figure 3:**
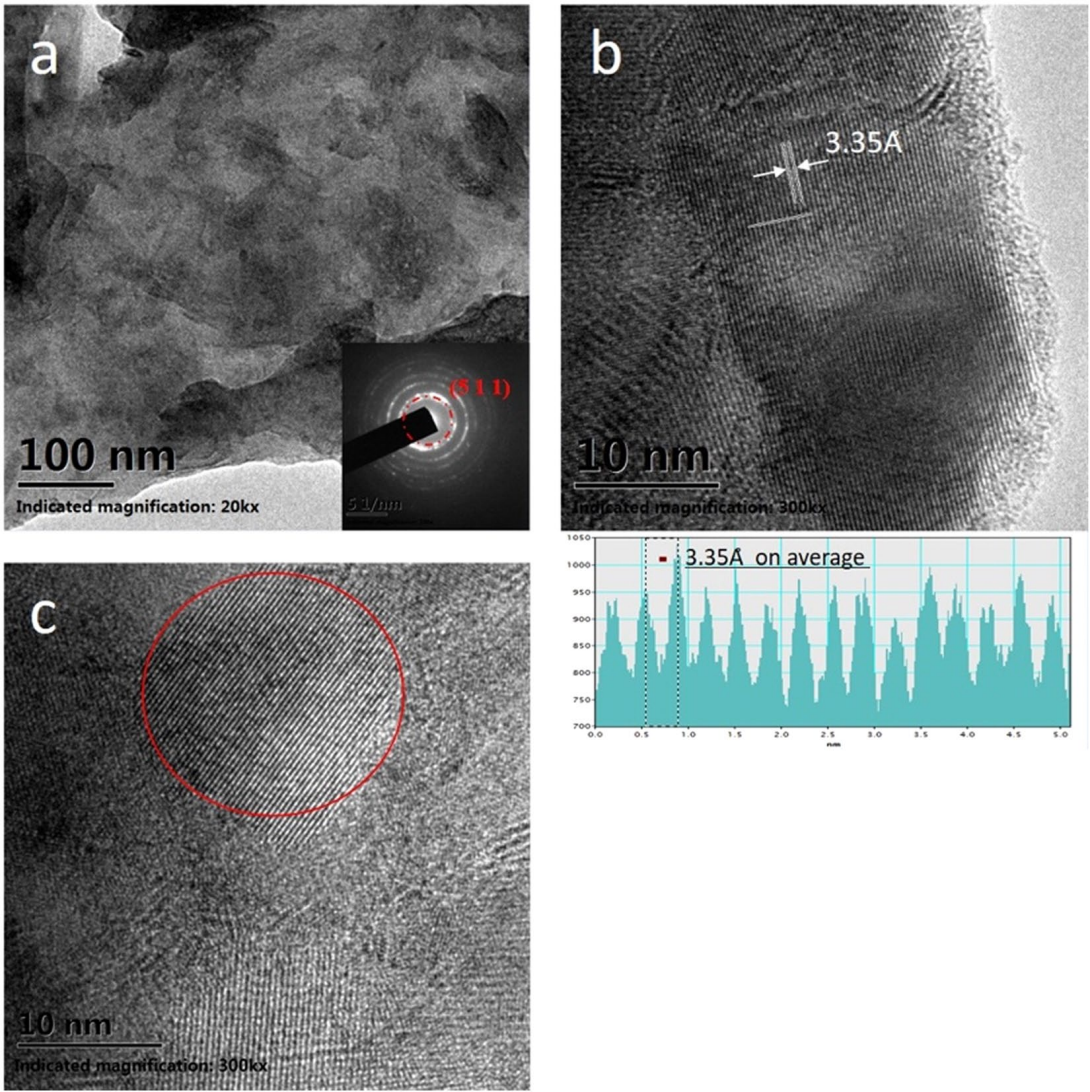
(**a**) Overview TEM images of the few layer In_2_Te_3_ and SAED patterns (inset). (**b**) Few layer In_2_Te_3_ combines the crystalline areas with lattice spacing of 3.35 Å and less crystalline areas. (**c**) The ordering phase in red circle interspaced by disordered phases.

To study the crystalline structures in more details, TEM are conducted for few-layer α-In_2_Te_3_. Figure 3 presents the low- and high-resolution TEM images having a typical polycrystalline structures. The electronic diffraction patterns of the few-layer polycrystalline In_2_Te_3_ can distinguish (511) plane with higher multitude than other diffraction rings in inset of Fig. 3a. As marked lines in Fig. 3b, the lattice spot measures reveals a inter plane spacing of 3.35 Å. It can be assigned to the (511) planes of α-In_2_Te_3_. As shown in the circled area of image Fig. 3b,c, highly ordered structure with high crystallization is surrounded by less crystallized areas with many defects or highly disordered structures. Such kind of defects and disorder will profoundly affect the electronic properties of few-layer In_2_Te_3_ as discussed below.

**Figure 4:**
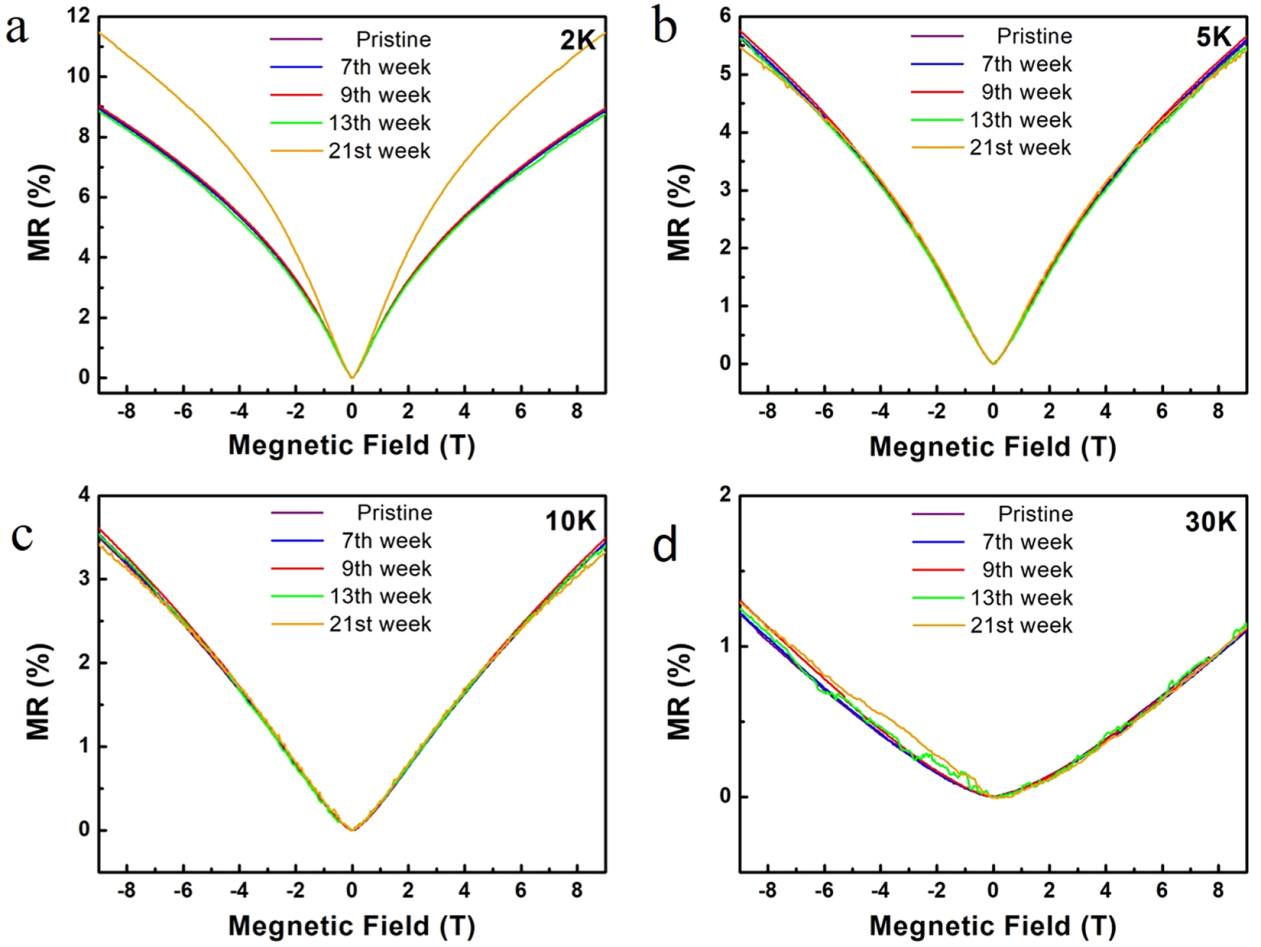
MR at θ = 90° with different exposure time in ambient air at (**a**) 2 K, (**b**) 5 K, (**c**) 10 K and (**d**) 30 K.

The MR properties of the few layer In_2_Te_3_ at different exposure in air was studied for the first time. Figure 4a–d show the MR of the few layer In_2_Te_3_ under the vertical magnetic field at 2, 5, 10 and 30 K respectively. MR was found to be constant at all the temperature and a slight increment in MR observed at 2 K up to 21 weeks. MR decreases with the increase of temperature and no obvious MR difference over different exposure time in air has been found for a certain temperature (Fig. 4b–d). Another MR observation of the In_2_Te_3_ few layers is that MR varies quadratically with increasing magnetic field at 2 K and 5 K due to the weak anti-location effect like WTe_2_ systems^41,42^ (Fig. 4a,b). The weak anti-location effect was kept under long air exposure even after 21 weeks (Fig. 4a,b). Such effect was disappeared at 10 K and 30 K because of the disappearing of the quantum interference at higher temperature, and a linear dependence of MR with magnetic field is restored (Fig. 4c,d). The stability of MR property in few layer In_2_Te_3_ was clearly observed during the long exposure in air in this stability test.

**Figure 5:**
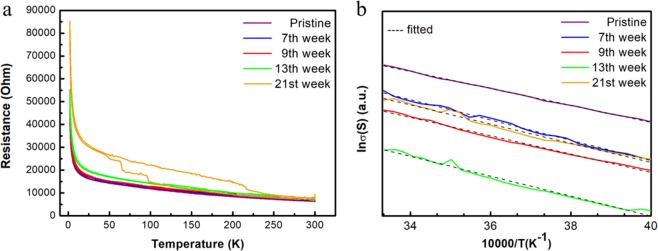
(**a**) Temperature dependence of the four terminal resistances in up to 21 weeks. (**b**) The barrier energy results in room temperature range for samples exposed different time in air.

We further study the electronic properties of the few layer In_2_Te_3_ by measuring the resistance at different temperature (Fig. 5a). The resistance has been increasing slightly at room temperature, with the exposure time increasing. But the resistance changes dramatically at low temperature specifically from 44217 Ohm for pristine sample to 85275 Ohm for 21 weeks exposed sample. The fitting of the lnσ vs. 10000/T, where σ is conductance and T is temperature, in Fig. 5b shows a barrier energy gap of ~0.23 eV. The result is consistent with the previous reports where disorder and defects in In_2_Te_3_ will reduce the band gap dramatically in In_2_Te_3_^43^.

In summary, we have developed a feasible method for large area few layer α-In_2_Te_3_ thin films via CVD techniques. The few layer In_2_Te_3_ shows the crystalline characteristics in most part with some disordered areas, which significantly influences the electronic properties. Positive MR property are studied for this 2D few layer semiconductor for the first time, and the largest transverse MR was observed up to 11% at 2 K. The MR in few layer α-In_2_Te_3_ thin films shows high stability in long exposure time in ambient air.

## Methods

### Preparation of Few-layer α-In_2_Te_3_ thin film

Few-layer α-In_2_Te_3_ thin film was deposited by CVD method onto a precursor substrate, which had been coated with ~2 nm thickness of In_2_O_3_ in resistance heating vacuum coating system. We used pure In_2_O_3_ powder 0.2~0.25 g and SiO_2_ (200 nm)/Si (100) wafer to fabricate these precursors. Before deposition of thin film, the SiO_2_ substrates were cleaned with alcohol in ultrasonic bath for 10 min three times then with acetone one time for 10 min to remove both inorganic and organic contaminations, respectively. The powder was put in tungsten boat and then heated under a pressure of 6.0 * 10^−3^ Pa to deposit onto substrates by thermal evaporation. As mentioned later, thermal evaporation of In_2_O_3_ plays important role in determining whether we can acquire In_2_Te_3_ thin films in the following subsequent CVD step. The deposition rate of thermal evaporation is not easy to precisely control. So we acquired In_2_O_3_ ultrathin films with homogeneous morphology monitored by an optical microscope after adjustment of parameters by increasing the heating power steadily. Then 0.26~0.3 g Te powder was used as source and was placed at the first thermal zone of an oven. The as-deposited In_2_O_3_were placed in another thermal zone at downstream of the carrier gas with a mixture of H_2_ and Ar (15 and 45 sccm, respectively). The evaporation temperature in first thermal zone is set over the melting temperature of Te and the reaction temperature at the substrate is limited by 440 °C at the second zone. Although In_2_O_3_ has a decomposition temperature around 1913 °C and melting temperature of In_2_Te_3_ is 667 °C, in the condition of larger and atomically-thin reaction surfaces, we have to reduce synthesis temperature in order to prevent product from evaporation. To eliminate contamination of O_2_, we evacuate quartz tube and refill it with N_2_ three times. Both evaporation and reaction area persist in overheating from room temperature to 500 °C and 440 °C within 20 min respectively and dwell for 80 min. The samples are cooled down to room temperature under reaction atmosphere.

### Characterizations

We used optical microscope (OM, MV6100) to select promising samples with integrity and uniformity. To authenticate elemental composition and chemical states of the deposited films, we used Raman spectroscopy (LabRAM HR800, Horiba JobinYvon) with an excitation laser of 532 nm, and X-ray photoelectron spectroscopy (XPS, AXIS-ULTRADLD-600W, Kratos) techniques. And investigation of thickness information was carried out by the atomic-force microscopy (AFM, SPM9700, Shimadzu). The crystallographic structure of In_2_Te_3_ films was examined by X-ray diffraction (XRD, Empyrean, PANalytical B.V.) as well as transmission electron microscope (TEM, JEM2100HR).Finally the transport properties were conducted by physical property measurement system (PPMS, Quantum Design) with a four-terminal configuration using silver electrodes and temperature range from 300 to 2 K. The electrodes were fabricated with the same area, shape, and the distance between neighbouring electrodes. For the MR measurements, the variable magnetic field was set as 40 Oe s^−1^ (1 T = 10000 Oe) under the vertical magnetic field at 2, 5, 10 and 30 K respectively. For the resistance measurements, the cooling rate was set as 2 K min^−1^ with the interval of ∼1 K. All the resistance measurements were carried out at a constant current mode.
